# The Effect of Neuronal CoQ_10_ Deficiency and Mitochondrial Dysfunction on a Rotenone-Induced Neuronal Cell Model of Parkinson’s Disease

**DOI:** 10.3390/ijms25126622

**Published:** 2024-06-16

**Authors:** Lauren Millichap, Nadia Turton, Elisabetta Damiani, Fabio Marcheggiani, Patrick Orlando, Sonia Silvestri, Luca Tiano, Iain P. Hargreaves

**Affiliations:** 1 Department of Life and Environmental Sciences, Polytechnic University of Marche, I-60131 Ancona, Italy; l.millichap@pm.univpm.it (L.M.); e.damiani@staff.univpm.it (E.D.); f.marcheggiani@univpm.it (F.M.); p.orlando@univpm.it (P.O.); s.silvestri@univpm.it (S.S.); l.tiano@staff.univpm.it (L.T.); 2 School of Pharmacy and Biomolecular Sciences, Liverpool John Moores University, Liverpool L3 5UA, UK; n.m.turton@2020.ljmu.ac.uk

**Keywords:** mitochondrial dysfunction, oxidative stress, coenzyme Q_10_, neurodegeneration, Parkinson’s disease, pentose phosphate pathway, mitochondrial biogenesis, antioxidant defences, therapeutics

## Abstract

Parkinson’s disease (PD) is the second most prevalent neurodegenerative disorder currently affecting the ageing population. Although the aetiology of PD has yet to be fully elucidated, environmental factors such as exposure to the naturally occurring neurotoxin rotenone has been associated with an increased risk of developing PD. Rotenone inhibits mitochondrial respiratory chain (MRC) complex I activity as well as induces dopaminergic neuronal death. The aim of the present study was to investigate the underlying mechanisms of rotenone-induced mitochondrial dysfunction and oxidative stress in an in vitro SH-SY5Y neuronal cell model of PD and to assess the ability of pre-treatment with Coenzyme Q_10_ (CoQ_10_) to ameliorate oxidative stress in this model. Spectrophotometric determination of the mitochondrial enzyme activities and fluorescence probe studies of reactive oxygen species (ROS) production was assessed. Significant inhibition of MRC complex I and II–III activities was observed, together with a significant loss of neuronal viability, CoQ_10_ status, and ATP synthesis. Additionally, significant increases were observed in intracellular and mitochondrial ROS production. Remarkably, CoQ_10_ supplementation was found to reduce ROS formation. These results have indicated mitochondrial dysfunction and increased oxidative stress in a rotenone-induced neuronal cell model of PD that was ameliorated by CoQ_10_ supplementation.

## 1. Introduction

**Figure 1:**
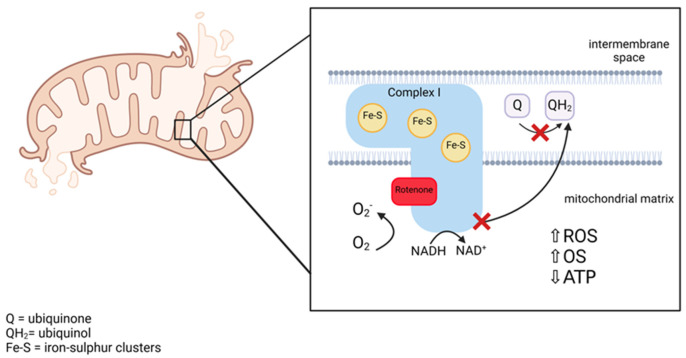
A schematic diagram to show the mechanism of rotenone-induced mitochondrial respiratory chain complex I inhibition, resulting in mitochondrial dysfunction and oxidative stress. [Created using biorender.com]. ROS—reactive oxygen species; OS—oxidative stress; ATP—adenosine triphosphate; O_2_—molecular oxygen; O_2_^−^—superoxide anion; NADH/NAD^+^—nicotinamide adenine dinucleotide.

Parkinson’s disease (PD) is the second most prevalent neurodegenerative disorder and the most common movement disorder affecting the current ageing population [1,2]. The clinical manifestations of the disease are characterised by a triad of motor symptoms (bradykinesia, rigidity, and tremor) and in the later stages, non-motor characteristics (cognitive dysfunction, depression, and behavioural and learning disorders) that increase in severity as the disease progresses, eventually leading to near total immobility [2,3,4]. PD is characterised by two main pathological features: loss of dopaminergic neurons within the substantia nigra pars compacta (SNpc) and the development of Lewy Bodies, predominately containing accumulated fibrillar α-synuclein [1,4]. Although the aetiology of PD has not yet been fully elucidated, PD is recognised as a multifactorial disease with both genetic and environmental risk factors contributing to disease development and progression [5]. However, the biggest risk factor for PD is increasing age, with the median age of onset being 60 years of age [5,6]. Although the aetiology of PD remains unknown, increasing evidence has demonstrated that impaired mitochondrial function and oxidative damage are significant aetiological factors in PD pathogenesis, resulting in alterations in the cellular redox state and injury to nervous system structural components, eventually leading to neuronal death [5,7]. Neurons are particularly susceptible to reactive oxygen species (ROS) accumulation and oxidative damage, as neurons rely on mitochondria due to their high metabolic demand because of inadequate glycolytic capacity [8,9,10]. The mitochondria are a major source of ROS as a by-product of oxidative phosphorylation (OXPHOS); therefore, increased oxidative stress caused by ROS accumulation as the result of impaired mitochondrial respiratory chain (MRC) function may result in the degeneration of dopaminergic neurons in PD [8,11]. The causes of MRC dysfunction in PD are as yet uncertain but may result from point mutations and deletions in the mitochondrial DNA of PD neurons, an impairment in iron metabolism affecting the assembly of the iron–sulphur prosthetic groups of the MRC, or the ability of α-synuclein to interact with the inner mitochondrial membrane and potentially inhibit the activity of the MRC enzymes [9]. Rotenone, a common agricultural pesticide, is known to induce Parkinsonian-like features by preferentially targeting dopaminergic neurons [12,13]. Rotenone is a neurotoxin capable of suppressing complex I (NADH-Ubiquinone oxidoreductase) of the MRC and can inhibit nicotinamide adenine dinucleotide (NADH) oxidation, leading to increased ROS production and OXPHOS impairments leading to adenosine triphosphate (ATP) deficiency (Figure 1) [12,14]. The link between selective dopaminergic death in PD and oxidative damage is further supported by pentose phosphate pathway (PPP) dysregulation [15]. The mechanism of action of rotenone involves the inhibition of electron transfer from the iron–sulphur (Fe-S) clusters located within complex I to ubiquinone by binding to the ubiquinone binding site located on complex I, resulting in mitochondrial dysfunction (Figure 1) [12,14,16]. Several rodent studies have reported Parkinsonism features following rotenone administration by daily intraperitoneal injections, and postmortem rat studies found a significant loss of substantia nigra and striatal neurons following rotenone treatment consistent with PD pathology [17].

The increased oxidative stress and mitochondrial dysfunction associated with PD may result from diminution in neuronal Coenzyme Q_10_ (CoQ_10_) content. CoQ_10_ is a small lipophilic molecule that is ubiquitous in all cell membranes, markedly the mitochondria, and forms a vital component of the MRC, in addition to serving as an important antioxidant, protecting cell membranes from ROS-induced oxidative stress [18,19]. Therefore, CoQ_10_ supplementation may provide a neuroprotective effect in PD by restoring endogenous CoQ_10_ levels in addition to ameliorating oxidative stress and mitochondrial dysfunction due to its antioxidant properties and its ability to restore electron flow in the MRC [20]. CoQ_10_ supplementation has shown some therapeutic neuroprotection in a rodent model of chemically induced Parkinsonism, including reduced mortality and decreased oxidative stress [21]. Moreover, in a study carried out by Attia et al. [22], therapeutic intervention with CoQ_10_ in mice prior to exposure to paraquat, an alternative PD-inducing pharmacological compound, was found to decrease oxidative stress biomarkers in the brain and protect against mitochondrial damage, demonstrating the potential neuroprotective effect of CoQ_10_ in slowing down the progression of PD [22]. However, although the mechanisms behind the therapeutic efficacy of CoQ_10_ supplementation are not yet elucidated, as a natural component, CoQ_10_ supplementation is well tolerated and safe for use as a dietary supplement and does not cause any serious adverse effects in humans or experimental animals [23]. Therefore, CoQ_10_ supplementation may be a potential therapeutic approach to scavenging free radicals and ameliorating oxidative-stress-induced impairments in PD patients.

The neuroblastoma SH-SY5Y cell line has been used extensively in neuroscience research and is a commonly used in vitro model of dopaminergic neurons in PD research [24,25]. Although this cell line exhibits a number of genetic abnormalities due to its cancerous origin, most genes and pathways that are dysregulated in PD remain intact. Mitochondrial dysfunction and oxidative stress are key events leading to the development of neurodegeneration in PD; therefore, in this study, the consequences of impaired mitochondrial function and limited cellular antioxidant status and the effect of elevated oxidative stress and ROS production on the SH-SY5Y neuronal cells were investigated following rotenone exposure. PPP dysregulation may be an early event in PD pathogenesis; therefore, the association between the effect of glucose-6-phosphate dehydrogenase (G6PDH) activity and reduced GSH was also investigated. In order to investigate the potential therapeutic mechanism of CoQ_10_ in the treatment of PD, the ability of CoQ_10_ (5 µM) supplementation to ameliorate cellular oxidative stress in a rotenone-induced neuronal cell model of PD was assessed [24].

## 2. Result

### 2.1. The Effect of Rotenone Treatment on Cell Viability

**Figure 2:**
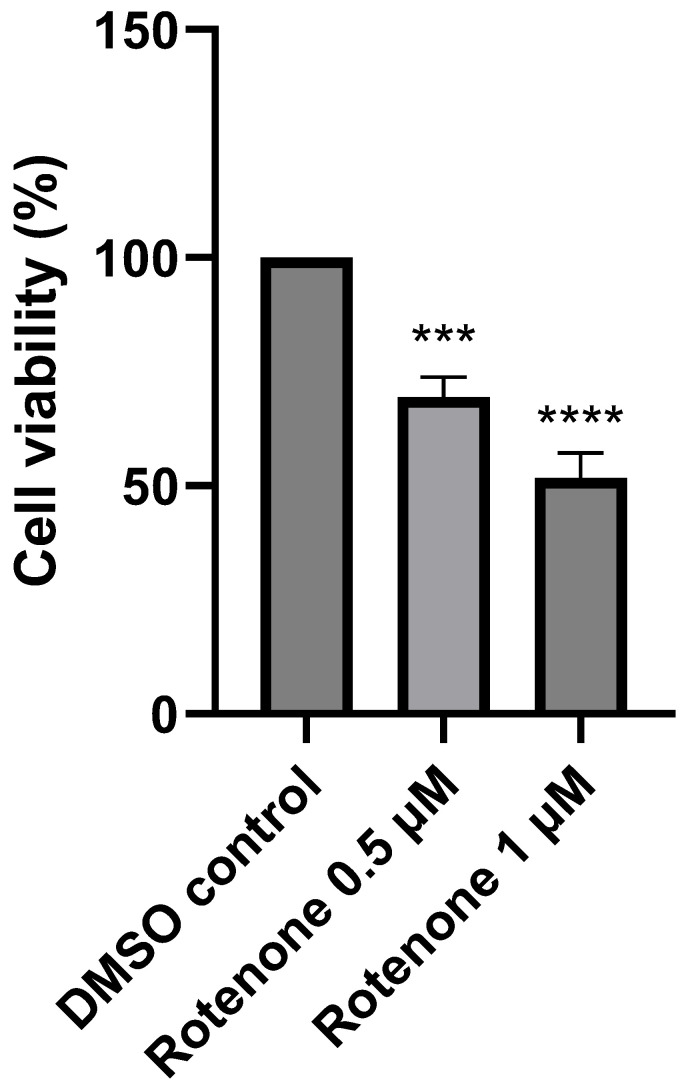
Cell viability (%) of SH-SY5Y human neuroblastoma cells following 24 h incubation with rotenone (0.5 µM and 1 µM). Error bars represent standard error of the mean (SEM); statistical analysis was carried out using One-Way ANOVA with Tukey’s multiple comparison post hoc test; levels of significance *** *p* < 0.0005, **** *p* < 0.0001 compared to control levels (n = 3). DMSO—dimethyl sulfoxide.

The cellular toxicity induced by rotenone treatment was found to be both time- and dose-dependent. Treatment with 0.25 µM rotenone had very little effect on cell viability, whereas 2 µM rotenone induced a significant reduction in cell viability (<50%). However, following treatment with 0.5 µM and 1 µM rotenone, cell viability was reduced by 31% (*p* < 0.0005, n = 3) and 48% (*p* < 0.0001, n = 3), respectively, compared to control cells treated with the vehicle dimethyl sulfoxide (DMSO) (Figure 2).

### 2.2. The Effect of Rotenone Treatment on Cellular ROS Production

**Figure 3:**
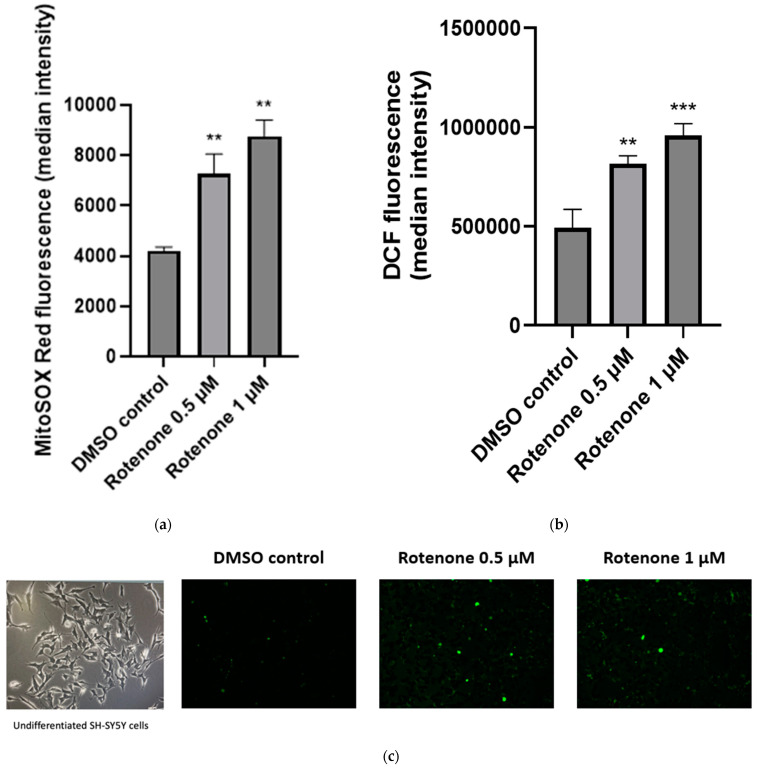

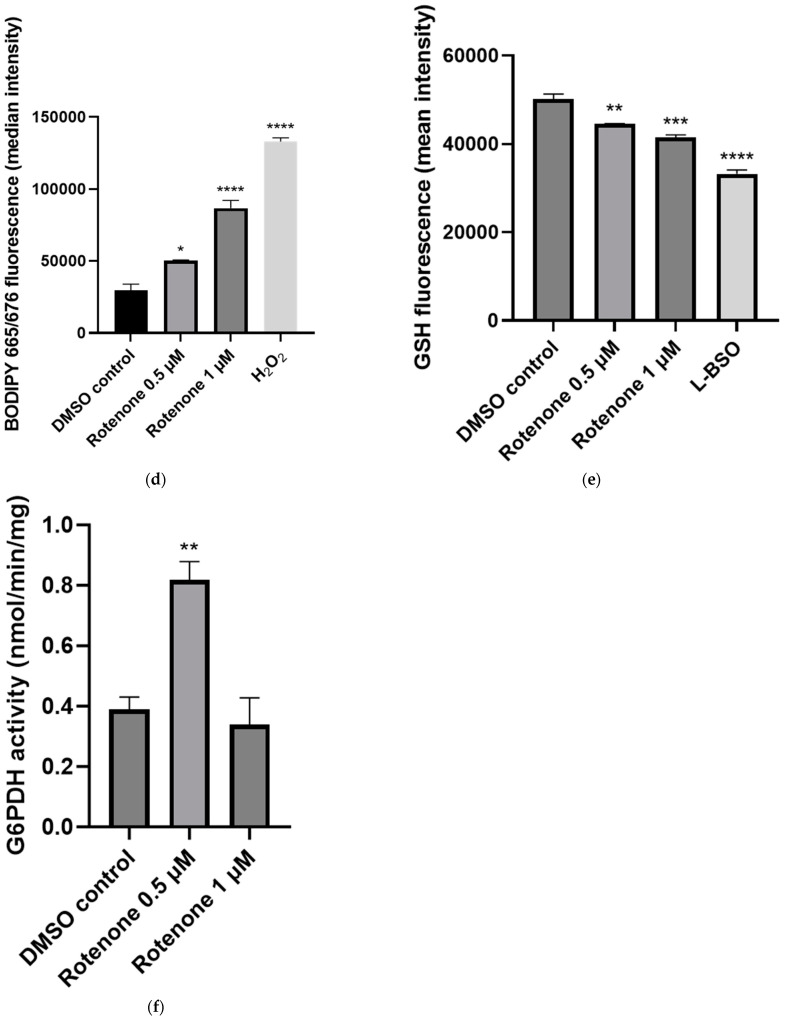
Effect of 24 h rotenone exposure (0.5 µM and 1 µM) on cellular reactive oxygen species (ROS) production. (**a**) Measurement of intracellular ROS production in SH-SY5Y human neuroblastoma cells following staining with CM-H_2_DCFDA. (**b**) Measurement of mitochondrial superoxide production in SH-SY5Y human neuroblastoma cells following SH-SY5Y staining with MitoSOX Red™. (**c**) Evaluation of intracellular ROS by CM-H_2_DCFDA assay in the SH-SY5Y human neuroblastoma cell line following rotenone treatment (0.5 μM and 1 μM) for 24 h. Representative images taken with a Lionheart Automated Microscope post-CM-H_2_DCFDA staining. (**d**) Assessment of lipid peroxidation in SH-SY5Y human neuroblastoma cells following SH-SY5Y staining with BODIPY™ 665/676. (**e**) Measurement of intracellular reduced glutathione (GSH) status in SH-SY5Y human neuroblastoma cells following SH-SY5Y staining with monochlorobimane (mBCl). (**f**) Measurement of glucose-6-phosphate dehydrogenase (G6PDH) activity in SH-SY5Y human neuroblastoma cells. The absorbance of G6PDH was measured at a wavelength of 450 nm. Error bars represent standard error of the mean (SEM); statistical analysis was carried out using One-Way ANOVA with Tukey’s multiple comparison post hoc test; levels of significance * *p* < 0.05, ** *p* < 0.005, *** *p* < 0.0005, **** *p* < 0.0001 compared to control levels (n = 3). DMSO—dimethyl sulfoxide; H_2_O_2_—hydrogen peroxide; L-BSO—L-Buthionine sulphoximine; G6PDH—glucose-6-phosphate dehydrogenase.

Following the treatment of neuronal cells with rotenone at concentrations of 0.5 µM and 1 µM, intracellular ROS production was found to be significantly increased by 66% (*p* < 0.005, n = 3) and 95% (*p* < 0.0005, n = 3), respectively, compared to control cells treated with the vehicle DMSO < 0.1% (*v*/*v*) (Figure 3b,c).

Mitochondrial superoxide anion production was also found to be significantly increased following treatment with rotenone (0.5 µM and 1 µM), increasing by 88% in the presence of 0.5 µM rotenone (*p* < 0.05, n = 3) and 100% in the presence of 1 µM (*p* < 0.005, n = 3) compared to control levels (Figure 3c). In addition, the treatment of neuronal cells with 0.5 µM and 1 µM rotenone induced a significant 69% (*p* < 0.05, n = 3) and 192% (*p* < 0.0005, n = 3) increase, respectively, in free-radical-induced lipid peroxidation (Figure 3d).

Cellular GSH status was found to be significantly decreased by 11% (*p* < 0.005, n = 3) and 17% (*p* < 0.0005, n = 3), respectively, compared to control cells treated with the vehicle DMSO following treatment with 0.5 µM and 1 µM rotenone (Figure 3e). In view of the decreased cellular GSH status, the effect of rotenone treatment on the activity of glucose-6-phopshate dehydrogenase (G6PDH), the rate-limiting enzyme of the oxidative phase of the PPP, was investigated [25]. Treatment of the neuronal cells with 0.5 µM rotenone resulted in a significant 52% (*p* < 0.005, n = 3) increase in G6PDH activity compared to control cells treated with the vehicle DMSO (Figure 3f). In contrast, treatment with 1 µM rotenone resulted in a non-significant (*p* < 0.8223) 13% decrease in GPDH activity (n = 3) (Figure 3f).

### 2.3. The Effect of Rotenone Treatment on Mitochondrial Function

**Figure 4:**
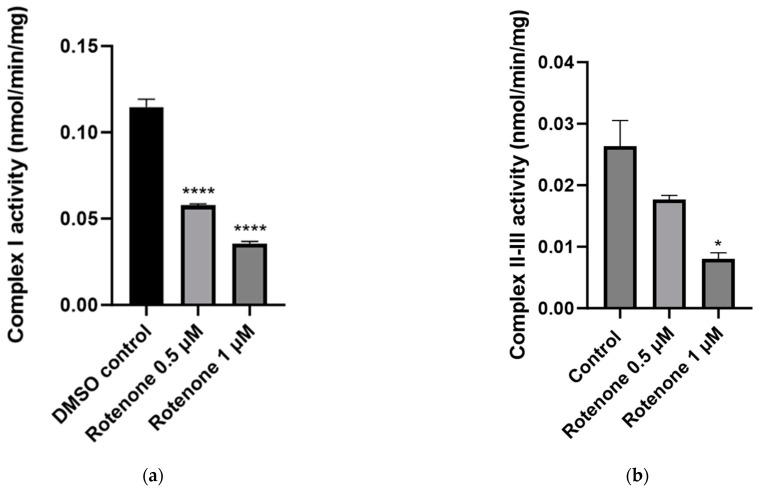

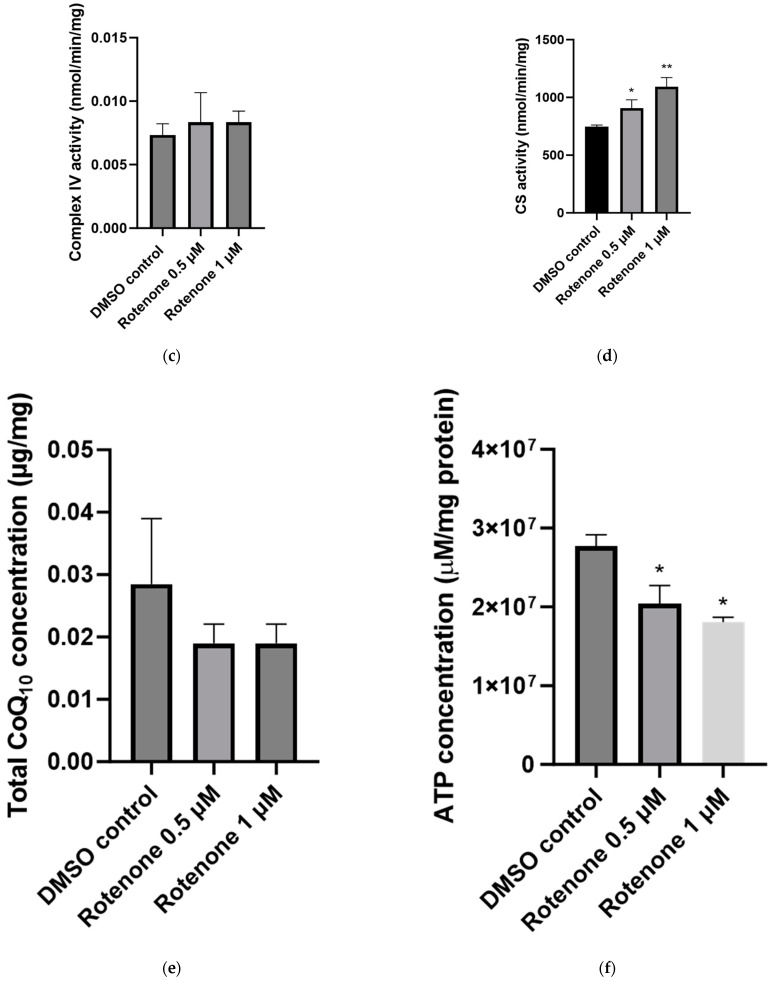
Effect of 24 h rotenone exposure (0.5 µM and 1 µM) on mitochondrial function in SH-SY5Y human neuroblastoma cells. (**a**) Measurement of complex I (NADH-Ubiquinone oxidoreductase) activity measured spectrophotometrically at a wavelength of 340 nm. (**b**) Measurement of complex II + III (succinate dehydrogenase cytochrome *c* reductase) activity measured spectrophotometrically at a wavelength of 550 nm. (**c**) Measurement of complex IV (cytochrome *c* oxidase) activity measured spectrophotometrically at a wavelength of 550 nm. (**d**) Measurement of citrate synthase (CS) activity measured spectrophotometrically at a wavelength of 412 nm. (**e**) Measurement of neuronal Coenzyme Q_10_ content (µg/mg) in SH-SY5Y human neuroblastoma cells. CoQ_10_ content of SH-SY5Y cells was determined using HPLC-ECD. (**f**) Measurement of intracellular adenosine triphosphate (ATP) concentration in SH-SY5Y human neuroblastoma cells. Intracellular ATP status was determined by ATP luminescence. Total cellular ATP concentration was expressed as a ratio to protein concentration (µM/mg); n = 3. Error bars represent standard error of the mean (SEM); statistical analysis was carried out using the Kruskal–Wallis test (Figure 3b) or One-Way ANOVA with Tukey’s multiple comparison post hoc test (Figure 3a,c–f); levels of significance * *p* < 0.05, ** *p* < 0.005, **** *p* < 0.0001 compared to control levels. DMSO—dimethyl sulfoxide; CoQ_10_—Coenzyme Q_10_; ATP—adenosine triphosphate.

Treatment of the SHS-5Y neuronal cells with 0.5 µM and 1 µM rotenone resulted in a significant 50% (*p* < 0.0001, n = 3) and 69% (*p* < 0.0001, n = 3) decrease, respectively, in MRC complex I activity compared to control cells treated with the vehicle DMSO (Figure 4a). In addition, treatment with 0.5 µM and 1 µM rotenone resulted in a 33% (non-significant; *p* < 0.5853, n = 3) and 70% (significant; *p* < 0.005, n = 3) decrease, respectively, in MRC complex II–III activity compared to control cells treated with the vehicle DMSO (Figure 4b). However, a non-significant (*p* < 0.8602) 12% increase in MRC complex IV activity was detected following treatment with rotenone (0.5 µM and 1 µM; Figure 4c). Rotenone treatment was also found to significantly increase the activity of the mitochondrial marker enzyme, CS, which increased by 18% (*p* < 0.05, n = 3) and 32% (*p* < 0.005, n = 3), respectively, compared to control cells treated with the vehicle DMSO following treatment with 0.5 µM and 1 µM rotenone (Figure 4d).

The CoQ_10_ content of SH-SY5Y neuronal cells was found to be decreased by 34% (*p* < 0.6521) following treatment with 0.5 µM rotenone. Subsequent treatment with 1 µM rotenone did not induce a further decrease in CoQ_10_ status (Figure 4e). The concentration of intracellular ATP was also found to be significantly decreased by 26% (*p* < 0.05, n = 3) and 35% (*p* < 0.05, n = 3), respectively, compared to control cells treated with the vehicle DMSO following treatment with 0.5 µM and 1 µM rotenone (Figure 4f).

### 2.4. The Effect of CoQ_10_ Supplementation on Rotenone-Treated Cells

**Figure 5:**
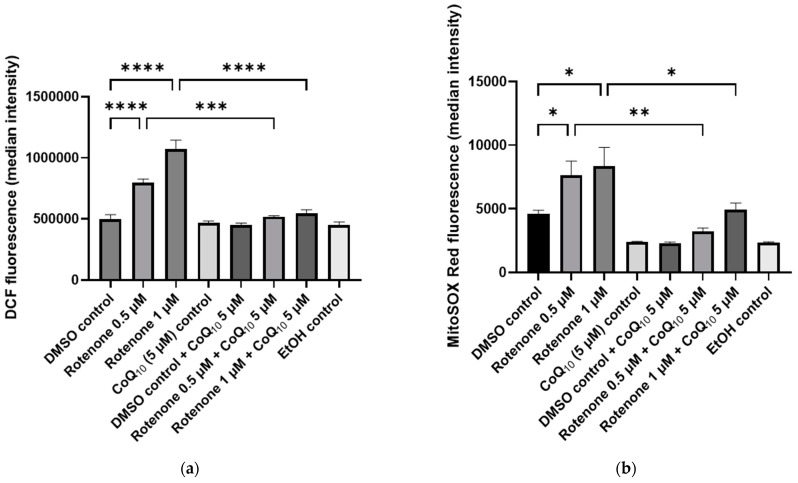
Effect on the ability of 3-day CoQ_10_ (5 µM) supplementation prior to 24 h rotenone exposure (0.5 µM and 1 µM) to prevent elevated cellular reactive oxygen species (ROS) production in SH-SY5Y human neuroblastoma cells determined by flow cytometry and indicated by median fluorescence intensity. (**a**) Measurement of intracellular ROS production in SH-SY5Y human neuroblastoma cells following SH-SY5Y staining with CM-H_2_DCFDA. (**b**) Measurement of mitochondrial superoxide production in SH-SY5Y human neuroblastoma cells following SH-SY5Y staining with MitoSOX Red™. Error bars represent standard error of the mean (SEM); statistical analysis was carried out using One-Way ANOVA with Tukey’s multiple comparison post hoc test; levels of significance * *p* < 0.05, ** *p* < 0.005, *** *p* < 0.0005, **** *p* < 0.0001 compared to control levels (n = 3). DMSO—dimethyl sulfoxide; CoQ_10_—Coenzyme Q_10_; EtOH—ethanol.

Pre-treatment of neuronal cells with CoQ_10_ for 3 days prior to rotenone (0.5 µM and 1 µM) treatment maintained ROS at untreated control levels (Figure 5b). Pre-supplementation with CoQ_10_ of 0.5 µM and 1 µM rotenone-treated neuronal cells induced a significant 35% (*p* < 0.0005, n = 3) and 49% (*p* < 0.0001, n = 3) decrease in ROS production, respectively, compared to rotenone-treated cells not pre-supplemented with CoQ_10_ (Figure 5b). Additionally, pre-supplementation with CoQ_10_ was also found to decrease mitochondrial superoxide production by 58% (*p* < 0.005, n = 3) and 41% (*p* < 0.05, n = 3), respectively, in neuronal cells treated with 0.5 µM and 1 µM rotenone compared to non-CoQ_10_ supplemented rotenone-treated cells (Figure 5b).

## 3. Discussion

The results of the present study have provided evidence of rotenone-induced elevated ROS levels, reduced intracellular CoQ_10_, and GSH status that was associated with impairments of MRC complex I and II–III activities. In addition, this study has also indicated the potential neuroprotective effect of CoQ_10_ and its ability to ameliorate oxidative stress in the SH-SYSY neuronal cell model of PD. Although the pathogenic mechanisms that underlie PD are unknown, oxidative damage and mitochondrial dysfunction have been well reported in PD pathogenesis, leading to the degeneration of dopaminergic neurons [1,25]. The SH-SY5Y human neuroblastoma cell line is a commonly used in vitro model for PD research, as these cells express numerous key proteins essential for dopaminergic neuronal function [26]. Furthermore, although rotenone exposure has been associated with decreased MRC complex I deficiency and elevated cellular oxidative stress [26,27], this is the first study to report evidence of secondary MRC complex II–III dysfunction and decreased CoQ_10_ status following neuronal exposure to rotenone, which may be important factors to consider in disease pathophysiology.

Elevated ROS levels were detected following rotenone exposure (0.5 µM and 1 µM), indicating the ability of rotenone to induce elevated levels of intracellular and mitochondrial ROS, measured by means of redox-sensitive fluorescent dyes, CM-H_2_DCFDA and MitoSOX™ Red. This study also identified an increase in rotenone-induced oxidative damage to cellular membranes indicated by fluorescence using the BODIPY™ 665/676 indicator, which was used to identify peroxyl radicals, an indicator of lipid peroxidation processes. Rotenone at a concentration of 1 µM was found to induce a profound elevation in ROS levels, suggesting an increase in electron leakage from the MRC with increasing rotenone concentration. Although the mechanism of elevated ROS production as a result of rotenone exposure remains elusive, elevated ROS levels associated with rotenone exposure may be caused as a result of rotenone-induced MRC complex I inhibition [28]. Incomplete electron transfer to molecular oxygen caused by rotenone selectively binding to complex I may result in the formation of ROS, therefore causing damage to mitochondrial structural components, which can eventually result in neuronal cell damage and apoptosis [29,30]. This may explain to some degree the evidence of the loss of cell viability (40–50% loss of viability) indicated by the MTT assay post-rotenone (0.5 µM and 1 µM) exposure. These results suggest that rotenone exposure to SH-SY5Y neuronal cells causes an elevation in oxidative stress by producing excess ROS.

Another possible explanation for the elevated ROS levels may be the antioxidant system becoming overwhelmed, therefore leading to increased ROS levels and contributing to neuronal cell damage and death [31]. The results of this study indicated that the intracellular GSH status was significantly depleted following rotenone exposure (0.5 µM and 1 µM) compared to control levels. Glutathione (GSH) is a major non-enzymatic antioxidant in the central nervous system (CNS); therefore, a reduction in both intracellular and mitochondrial GSH levels can lead to increased oxidative stress and impaired mitochondrial function via a selective reduction in the activity of MRC complex I, suggested to occur by a NO-mediated mechanism [32,33,34]. It has been suggested that total GSH depletion may occur before impaired mitochondrial function, particularly decreased MRC complex I activity and reduced dopamine levels, which may contribute to a loss of cell viability and progressive cell death [32,35]. Interestingly, a loss of total GSH levels is one of the earliest biochemical abnormalities observed in PD patients, prior to the selective loss of complex I activity, and has been suggested to contribute to subsequent dopaminergic cell death in PD [32,33,36].

Given that rotenone exposure results in MRC complex I deficiency, the effect of rotenone exposure on the activities of complex I, complex II–III, and complex IV was assessed. Interestingly, these results support the evidence that rotenone can suppress complex I of the MRC, as this study shows that rotenone exposure (0.5 µM and 1 µM) in SH-SY5Y cells results in a significant loss of complex I activity. Notably, the activity of complex I was reduced by 50%, as observed in cells treated with 0.5 µM rotenone, and 69% in cells treated with 1 µM rotenone, which may be sufficient enough to impair OXPHOS, as it has been previously reported that in order to compromise OXPHOS and ATP synthesis, complex I activity has to be severely impaired with a minimum reduction of 25% in activity [37]. This may explain the reduction in the intracellular ATP status observed in the SH-SY5Y cells following rotenone exposure, as indicated by the ATP luminescence assay, and where rotenone toxicity resulted in a 25–35% inhibition of ATP synthesis. Furthermore, a loss of ATP may explain the loss of cell viability, as neuronal glycolysis cannot compensate for the loss of mitochondrial ATP synthesis; therefore, a 35% inhibition of OXPHOS may compromise neuronal energy homeostasis in the synapse [37,38]. Additionally, rotenone-induced MRC complex I inhibition causing incomplete electron transfer within the MRC can lead to ATP depletion, therefore inducing oxidative stress and apoptosis [29].

The effect of rotenone exposure was also assessed on the activities of complex II–III and complex IV. The results from this study show that complex II–III activity is subsequently suppressed following rotenone exposure, where the activity of complex II–III was reduced by 33% and 70%, respectively. However, it has been previously reported that the inhibition threshold for complex III activity is 70–80% before OXPHOS is compromised [37]. These results are clear evidence of the mitochondrial dysfunction that may arise from rotenone-induced oxidative stress, supported by evidence of increased levels of oxidative by-products. The MRC generates ROS, mainly at complex I and complex III; however, there is evidence to support that complex II also contributes to the ROS pool [39]. Interestingly, these results show that complex IV activity was increased post rotenone exposure in SH-SY5Y cells. It has been reported that increased complex IV activity may occur as a result of specific neuronal overexpression of peroxisome proliferator-activated receptor-gamma (PPAR) coactivator 1-alpha (PGC1α) elevated mitochondrial biogenesis, leading to increased numbers of mitochondria and complex IV activity [40]. The significant increase in CS activity following treatment with rotenone would indicate that rotenone induces an increase in mitochondrial biogenesis in SH-SY5Y cells, which has been reported to occur as an adaptive response to an OXPHOS deficit [41]. The activities of the MRC complexes were expressed as a ratio to CS to account for mitochondrial enrichment; therefore, these results suggest that a reduction in the activities of the MRC complexes indicate impairment to OXPHOS rather than a loss of mitochondrial number [42].

A deficit in cellular CoQ_10_ content was identified post-rotenone exposure in SH-SY5Y cells (non-significant results), proposing the ability of rotenone to induce neuronal CoQ_10_ deficiency. CoQ_10_ is essential for normal mitochondrial function, and it has been reported that CoQ_10_ deficiency can lead to increased ROS generation, reduced ATP synthesis, and apoptosis induction [19]. The cause of cellular CoQ_10_ deficiency associated with rotenone toxicity has not yet been elucidated; however, the cause of a deficit in neuronal CoQ_10_ content may be caused by the ability of rotenone to directly inhibit enzymes involved in the CoQ_10_ biosynthetic pathway or as a result of an alternative secondary mechanism. Furthermore, a deficit in neuronal CoQ_10_ content was accompanied by a loss of complex II–III activity in comparison to control levels, suggesting that a reduction in complex II–III activity is indicative of a CoQ_10_ deficit, as the activity of these enzymes are directly dependent on endogenous CoQ_10_ availability [43].

Alterations in the activity of G6PDH were observed following rotenone exposure in SH-SY5Y cells. At the lowest concentration of rotenone (0.5 µM), a significant elevation in the activity of G6PDH was observed compared to control levels, which may be as a result of an adaptive response to protect against oxidative-stress-induced cell damage and death [44,45]. G6PDH is considered an antioxidant enzyme due to its role in regenerating nicotinamide adenine dinucleotide phosphate (NADPH), required for the regeneration of GSH to detoxify ROS; therefore, G6PDH-derived NADPH may have a significant impact on the cellular oxidative stress damage response [46,47]. Previous reports have demonstrated that mice overexpressing G6PDH are protected against the loss of dopaminergic neurons and may provide protection against Parkinsonism through the production of PPP-generated NADPH [15,48]. Additionally, at an increased concentration of rotenone (1 µM), the activity of G6PDH was reduced by 13% in comparison to control levels (non-significant result), suggesting that a profound loss of cell viability must occur in order to detect a loss of G6PDH activity. It has been previously reported in nucleated cells that G6PDH inhibition enhances cellular oxidative stress and induces apoptosis [46]. As the PPP is the main source of defence against oxidative stress in neurons, inhibition of the PPP is deleterious to neurons [15,49,50]. It has been reported by Dunn et al. [15] that the downregulation of the PPP in PD may be a primary event in disease progression, and mitochondrial damage may occur as a direct result of PPP dysregulation, as postmortem human brain tissue showed a reduction in the activity of G6PDH in the putamen of early-stage PD and in the cerebellum of early- and late-stage PD, which may be causing increased levels of oxidative stress. Further investigation into potential mechanisms that underlie the alterations in the activity of G6PDH are required to understand the role of impaired mitochondrial function and increased oxidative stress in PD pathogenesis as a result of PPP dysregulation.

Interestingly, CoQ_10_ supplementation (in its oxidised, ubiquinone form) as a therapeutic treatment to prevent rotenone-induced neuronal damage was able to significantly restore intracellular ROS levels similar to that of the controls following treatment with rotenone (0.5 µM and 1 µM). SH-SY5Y cells were treated with the oxidised ubiquinone form of CoQ_10_ due to the reported improved bioavailability of some commercial formulations of the ubiquinone form of CoQ_10_ compared to that of ubiquinol (reduced form of CoQ_10_) [51]. Although the exact mechanism by which exogenous CoQ_10_ induces this effect is unknown, CoQ_10_ may protect SH-SY5Y cells against rotenone-induced oxidative stress as a result of its ability to act as an antioxidant and free radical scavenger, therefore preventing ROS-induced oxidative damage [52]. However, the failure of CoQ_10_ supplementation to restore intracellular ROS production to below control levels in SH-SY5Y cells following exposure to rotenone may indicate that the elevation in intracellular ROS may have occurred as a result of an alternative inhibitory mechanism. As previously stated, the activity of MRC complex I was significantly reduced by 69% in cells treated with 1 µM of rotenone, which may be sufficient to impair OXPHOS. Therefore, this may provide an explanation as to why CoQ_10_ supplementation (5 µM) was unable to restore mitochondrial ROS levels to below control levels, as OXPHOS may still be compromised within these cells [39]. Additionally, the failure of CoQ_10_ supplementation to restore mitochondrial ROS levels to below control levels may be due to the dosage of CoQ_10_ used in this study (5 µM). In agreement with this, Duberley et al. [39] reported that a dosage of 10 µM may be required to reach therapeutic concentrations in cell cultures and to restore MRC function. However, quantification of the MRC complex activities would be required in order to confirm this hypothesis. Furthermore, although ROS have been frequently attributed to cellular damage, they have an essential role in physiological signalling pathways; therefore, a significant reduction in ROS production below control levels may lead to detrimental effects on cellular function [39].

CoQ_10_ supplementation was also effective at significantly reducing mitochondrial oxidative stress to below control levels prior to treatment with rotenone (0.5 µM) in SH-SY5Y cells. ROS are mainly produced in the mitochondria under physiological conditions and are potent producers of cellular superoxide from complexes I and III of the MRC [8,53]. Mitochondrial superoxide is the proximal radical produced during oxidative stress within mitochondria and may be the major cause of cellular oxidative damage underlying ageing and neurodegenerative disorders [54,55]. The significant reduction in mitochondrial ROS production to below control levels following CoQ_10_ supplementation may be as a consequence of the antioxidant ability of CoQ_10_ and its ability to scavenge superoxide radicals, which has been indicated in other studies [55,56,57]. In a study undertaken by Pham et al. [56], CoQ_10_ supplementation was found to be effective at lowering mitochondrial hydrogen peroxide (H_2_O_2_) levels, suggesting that supplementation with CoQ_10_ can increase the efficiency of mitochondrial electron handling, therefore reducing electron leakage from the MRC and decreasing ROS-induced oxidative stress.

Additionally, several clinical studies have observed the therapeutic response of CoQ_10_ supplementation in PD patients. In a phase II clinical trial, oral CoQ_10_ supplementation was found to reduce functional decline in patients with early-onset PD [58]. Each patient was randomly assigned to receive CoQ_10_ (300, 600, or 1200 mg/day) or placebo. This study found that CoQ_10_ was safe and well-tolerated at the reported dosages, and that the highest CoQ_10_ dosage (1200 mg/day) was associated with a significant reduction in the progression of PD. However, although the mechanisms behind the therapeutic efficacy to CoQ_10_ supplementation are not yet elucidated, as a natural component, CoQ_10_ supplementation is well tolerated and safe for use as a dietary supplement and does not cause any serious adverse effects in humans or experimental animals [23]. Therefore, CoQ_10_ supplementation may be a potential therapeutic approach to scavenge free radicals and ameliorate oxidative-stress-induced impairments in PD patients, although methods to enhance the transport of exogenous CoQ_10_ across the blood–brain barrier are still required.

## 4. Materials and Methods

### 4.1. Chemicals

#### All Reagents Were of Analytical Grade

The following were purchased from Sigma Aldrich (Poole, UK).

The sterile cell culture products are as follows: Dulbecco’s Modified Eagles Medium (DMEM) high glucose, with L-glutamine and sodium pyruvate; foetal bovine serum, heat inactivated (EU approved); Trypsin-EDTA (0.25%), phenol red; sterile 0.4% Trypan blue solution; Penicillin-Streptomycin; PBS (Phosphate-Buffered Saline) tablets; dimethyl sulphoxide; rotenone ≥ 95%; potassium phosphate, monobasic (KH_2_PO_4_), 99+%, pure; potassium phosphate, dibasic (K_2_HPO_4_), 99+%, pure; magnesium chloride hexahydrate (MgCl_2_·6H_2_O), 99%; Trizma-base (reagent grade); BSA (Bovine Serum Albumin) ≥ 96%; Coenzyme Q_1_~95%; β-nicotinamide adenine dinucleotide, reduced disodium salt hydrate ≥ 97%; ethylenediaminetetraacetic acid (EDTA), ACS reagent, ≥99%; cytochrome *c* from equine heart ≥ 95%; potassium cyanide (KCN), ACS reagent, ≥96%; sodium succinate dibasic hexahydrate ReagentPlus^®^ ≥ 99%; antimycin A from *Streptomyces* sp.; Triton X-100; concentrated hydrochloric acid (HCl) solution; acetyl coenzyme A sodium salt ≥ 93%; oxaloacetic acid ≥ 97%; L-ascorbic acid, cell culture tested; 5,5′-Dithiobis(2-nitrobenzoic acid) (DTNB) ≥ 98%; sodium bicarbonate (NaHCO_3_), ACS reagent, ≥99%; L-Buthionine-sulfoximine, ≥97%.

The following were purchased from Invitrogen Ltd. (Invitrogen; Paisley, UK): MTT (3-(4,5-Dimethylthiazol-2-yl)-2,5-Diphenyltetrazolium Bromide); CM-H_2_DCFDA (General Oxidative Stress Indicator); MitoSOX Red™ mitochondrial superoxide indicator; BODIPY™ 665/676 (Lipid Peroxidation Sensor); Monochlorobimane (mBCl).

The CellTiter-Glo^®^ 2.0 Cell Viability Assay Kit was purchased from Promega (Promnega; Chilworth, UK).

The Glucose 6 Phosphate Dehydrogenase Assay Kit (Colormetric) (ab102529) was purchased from Abcam (Abcam; Cambridge, UK).

DC total protein assay Reagent A and Reagent B were purchased from Bio-Rad Laboratories Ltd. (Bio-Rad; Hemel Hempstead, UK).

### 4.2. Cell Culture

The SH-SY5Y human neuroblastoma cell line, derived from SK-N-SH, was cultured in Dulbecco’s Modified Eagle Medium (DMEM) supplemented with 1% penicillin and streptomycin and 10% fatal bovine serum (FBS). Passages 14–24 were used. Cultures were maintained at 37 °C in an incubator containing 5% CO_2_. SH-SY5Y cells were grown to 70–80% confluence before they were seeded for treatment and analysis. Upon reaching confluency, cells were harvested and washed twice with warm phosphate buffered saline (PBS), followed by the addition of warm trypsin. Cells were counted using Trypan blue dye solution and dye excluding cells were counted using a haemocytometer. Cells were seeded in plastic 96-well plates at a density of 40,000 cells/well or T-75 flasks at a density of 400,000 cells/mL prior to treatment with rotenone.

### 4.3. Rotenone Exposure

Rotenone was prepared by dissolving it in 100% dimethyl sulfoxide (DMSO), and the final concentration of DMSO was <0.1% (*v*/*v*), which showed no evidence of toxicity on these cells. Control cells were treated with the vehicle DMSO < 0.1% (*v*/*v*). Cell treatment with rotenone was carried out in DMEM at concentrations of 0.5 µM and 1 µM, and these were treated for 24 h with rotenone. SH-SY5Y cells were exposed to 0, 0.25, 0.5, 1, and 2 µM rotenone for 24 h at 37 °C for the rotenone toxicity evaluation. These concentrations of rotenone were selected based on the concentrations of rotenone reported to induce neurotoxicity in an SH-SY5Y PD cell model in a study undertaken by Han et al. [59]. The experimental conditions and the final concentrations of 0.5 and 1 µM rotenone used in the present study were then selected based on cell viability > 50% indicated by the MTT assay. 

### 4.4. CoQ_10_ Supplementation

To assess the effects of CoQ_10_ supplementation, the SH-SY5Y cells were pre-treated with 5 µM CoQ_10_, in its oxidised form, for 3 days. Prior to rotenone treatment, SH-SY5Y cells were washed with PBS and subsequently treated with rotenone for 24 h. This concentration of CoQ_10_ was selected based on the biochemical efficacy of this concentration in CoQ_10_-deficient fibroblasts in a study carried out by Lopez et al., where it was reported to ameliorate oxidative stress at this concentration [60]. CoQ_10_ was solubilised in ethanol due to the lipophilic nature of the compound, and then this ethanolic solution was incubated in DMEM at 37 °C for a few minutes and vortexed prior to addition to the SH-SY5Y cells. The ability of CoQ_10_ supplementation to prevent oxidative-stress-induced neuronal impairments to potentially prevent the development of neuronal degeneration in Parkinson’s disease was assessed in the SH-SY5Y cells. In order to assess the ability of CoQ_10_ to prevent oxidative-stress-induced neuronal impairments, SH-SY5Y cells were incubated at 37 °C with CoQ_10_ (5 µM) for 3 days followed by the addition of rotenone (0.5 µM and 1 µM) for 24 h. Control cells were treated with ethanol.

### 4.5. Cell Viability Assay

The cell viability of SH-SH5Y cells was assessed using the tetrazolium dye MTT (3-(4,5-dimethylthiazol-2-yl)-2,5-diphenyl tetrazolium bromide) assay (Invitrogen, UK). This colorimetric assay is used as a function of redox potential and is based on the ability of the mitochondrial NADPH-dependent cellular oxidoreductase enzymes to reduce water-soluble MTT to its insoluble purple formazan [61]. The amount of formazan produced is directly proportional to the number of live cells present following MTT exposure. SH-SH5Y cells were seeded and treated with rotenone as previously described. The MTT solution (20 µL) was added to each well of a 96-well plate after the 24 h rotenone treatment. Following a 3–4 h incubation with MTT at 37 °C, 100 µL of DMSO was added to each well to solubilise the formazan product, and the absorbance was read at a wavelength of 570 nm using a Tecan Spark Microplate Reader (Tecan; Zurich, Switzerland).

### 4.6. Intracellular ROS Measurement

Total cellular ROS levels were assessed in the SH-SY5Y cells using the general oxidative stress indicator, CM-H_2_DCFDA (Invitrogen; Paisley, UK). CM-H_2_DCFDA is a chloromethyl derivative of H_2_DCFDA and was used due to increased retention in live cells in comparison to H_2_DCFDA [62]. CM-H_2_DCFDA passively diffuses into live cells where intracellular esterases cleave its acetate groups to release its chloromethyl group, which is thiol-reactive, and subsequently reacts with thiols, including intracellular glutathione (GSH). Successive oxidation of the probe generates a fluorescent product trapped within the cells. It is understood that the fluorescent intensity of the cells is directly proportional to the ROS levels generated within the cells.

SH-SH5Y cells were seeded and treated with rotenone as previously described. A stock solution of CM-H_2_DCFDA (1 mM) was prepared by dissolving it in DMSO. On the day of the experiment, a final concentration of 1 µM CM-H_2_DCFDA was prepared in PBS. The media was removed from the 96-well plate post-rotenone treatment, and cells were washed twice with warm PBS before adding warm Trypsin (50 µL) and incubated for 5 min. Upon cell trypsinisation, DMEM (150 µL) was added, and the contents of the wells were transferred into Eppendorf tubes. The cells were centrifuged for 5 min at 500 rcf and the supernatant was discarded. An amount of 200 µL of the staining solution containing CM-H_2_DCFDA was added to the cell pellets, mixed both via pipetting and vortexing, and incubated for 15 min at 37 °C. The fluorescence intensity was measured via flow cytometry using the FL-1 channel (maximum excitation and emission spectra of 488 nm and 525 nm, respectively) on a BD Accuri™ C6 Flow Cytometer (BD Biosciences; Berkshire, UK).

### 4.7. Mitochondrial Superoxide Measurement

Mitochondrial superoxide production was assessed in SH-SY5Y cells using the mitochondrial superoxide indicator MitoSOX™ Red (Invitrogen; Pailsey, UK). MitoSOX™ Red is a fluorogenic dye used for the highly selective detection of mitochondrial superoxide in live cells and displays red fluorescence upon oxidation of the MitoSOX™ fluorescent probe [63]. MitoSOX™ Red is permeant to live cells and is rapidly and selectively targeted to the mitochondria. Upon entry into the mitochondria, the MitoSOX™ Red reagent is readily oxidised by superoxide and generates a highly fluorescent probe upon binding to nucleic acids. MitoSOX™ Red is specific to superoxide only and will not be oxidised by other ROS- or RNS-generating systems, prevented by superoxide dismutase (SOD). It is understood that the fluorescent intensity of the cells is directly proportional to the amount of mitochondrial superoxide generated within the cells.

SH-SY5Y cells were seeded and treated with rotenone as previously described. A stock solution of MitoSOX™ Red (5 mM) was prepared by dissolving it in DMSO. On the day of the experiment, a final concentration of 5 µM MitoSOX™ Red was prepared in PBS. The cells were prepared for flow cytometry as previously described. An amount of 200 µL of staining solution, containing MitoSOX™ Red, was added to the cell pellets, mixed both via pipetting and vortexing and incubated for 20 min at 37 °C. The fluorescence intensity was measured via flow cytometry using the FL-3 channel (maximum excitation and emission spectra of 500 nm and 582 nm, respectively) on a BD Accuri™ C6 Flow Cytometer (BD Biosciences, UK)

### 4.8. MRC Enzyme Activities

The activities of the MRC complexes and citrate synthase (CS) were determined spectrophotometrically on a Uvikon XS UV-Visible Scanning Spectrophotometer (NorthStar Scientific Ltd.; Bedfordshire, UK) [64]. The activities of MRC complex I (NADH-Ubiquinone oxidoreductase), complex II–III (succinate dehydrogenase cytochrome *c* reductase), complex IV, and CS were measured at wavelengths of 340 nm, 550 nm, and 412 nm, respectively, according to methods described by Duberley et al. [39]. The activity of complex IV is expressed as a first-order rate constant. The activities of the MRC complexes were expressed as a ratio to CS activity to equate to mitochondrial enrichment [42]. The activity of CS was expressed as a ratio to protein using the method by Lowry et al. [65].

### 4.9. Lipid Peroxidation Measurement

Lipid peroxidation (LPO) was assessed in SH-SY5Y cells using BODIPY™ 665/676 (Invitrogen; Pailsey, UK), an LPO sensor. BODIPY™ 665/676 is a lipophilic radical-sensitive fluorescent probe used for the detection of radical-driven lipid autoxidation [66]. Subsequent accumulation in the cellular membrane results in the dye becoming oxidised upon interaction with free radicals, including hydroxyl (OH^•^), alkoxyl (RO^•^), and peroxyl radicals (ROO^•^) [66,67]. BODIPY™ 665/676 demonstrates a change in fluorescence upon interaction, particularly with peroxyl radicals. It is understood that the fluorescent intensity of the cells is directly proportional to the amount of lipid peroxyls generated within the cells.

SH-SY5Y cells were seeded and treated with rotenone as previously described. A stock solution of BODIPY™ 665/676 (2.2 mM) was prepared by dissolving it in DMSO. On the day of the experiment, a final concentration of 10 µM BODIPY™ 665/676 was prepared in PBS. The cells were prepared for flow cytometry as previously described. An amount of 200 µL of staining solution containing BODIPY™ 665/676 was added to the cell pellets, mixed both via pipetting and vortexing, and incubated for 15 min at 37 °C. The fluorescence intensity was measured via flow cytometry using the FL-4 channel (maximum excitation and emission spectra of 665 nm and 676 nm, respectively) on a BD Accuri™ C6 Flow Cytometer (BD Biosciences; Berkshire, UK). Hydrogen peroxide (H_2_O_2_)-induced lipid peroxidation was used as a positive control for this experiment to ensure assay reliability.

### 4.10. Reduced Glutathione Measurement

Reduced glutathione (GSH) content was assessed in SH-SY5Y cells using the monochlorobimane (mBCl) fluorometric assay (Invitrogen; Paisley, UK). mBCl is non-fluorescent until it conjugates with low-molecular-weight thiols, including glutathione, N-acetylcysteine, and plasma thiols [68]. The glutathione conjugate of mBCl has a maximum excitation and emission spectra of 360 nm and 460 nm, respectively.

SH-SY5Y cells were seeded in opaque, black 96-well plates and treated with rotenone as previously described. A stock solution of mBCl (800 µM) was prepared by dissolving in PBS. On the day of the experiment, a working solution of 40 µM mBCl was prepared in PBS. To each well, 5 µL of mBCl (40 µM) was added to 95 µL DMEM. The cells were gently mixed and incubated for 30 min at 37 °C. The fluorescence intensity was measured at Ex = 360 nm and Em = 460 nm using a Tecan Spark Microplate Reader (Tecan; Zurich, Switzerland), according to the methods described by Sebastia et al. [69]. The total intracellular GSH concentration was expressed as a ratio to protein concentration (µM/mg protein). SH-SY5Y cells were treated with L-Buthionine sulphoximine (L-BSO) as a positive control to ensure the assay was able to detect a deficiency in GSH status. L-BSO is an inhibitor of gamma-glutamylcysteine synthetase (γ-GCS) and is known to deplete tissue GSH concentrations [70].

### 4.11. Intracellular ATP Measurement

Cellular adenosine triphosphate (ATP) luminescence was assessed in SH-SY5Y cells using a CellTiter-Glo^®^ Cell Viability Assay kit (Promega; Chilworth, UK). The CellTiter-Glo^®^ Assay is used as an indicator of metabolically active cells by determining the number of viable cells in culture based on quantitation of the ATP present [71]. This assay measures free intracellular ATP from viable cells to generate photons of light (bioluminescence) [71,72]. Viable cells are lysed to release intracellular ATP leading to the enzyme luciferase catalysing the mono-oxygenation of luciferin in the presence of Mg^2+^, ATP, and O_2_. This reaction yields oxyluciferin in an electronically excited state and CO_2_. Oxyluciferin in the excited state returns to the ground state, releasing a green-yellow luminescent light which can be detected using a luminometer. The intensity of the luminescent signal is directly proportional to the cellular ATP concentration.

SH-SY5Y cells were seeded in opaque, white 96-well plates and treated with rotenone as previously described. The method was followed according to the manufacturer’s instructions. The luminescence was recorded using a Tecan Spark Microplate Reader luminometer (Tecan; Zurich, Switzerland). The total intracellular ATP concentration was expressed as a ratio to protein concentration (µM/mg protein).

### 4.12. G6PDH Enzyme Activity

The activity of glucose-6-phosphate dehydrogenase (G6PDH) was assessed in SH-SY5Y cells using the Glucose 6 Phosphate Dehydrogenase Assay Kit (Colorimetric) (ab102529, Abcam; Cambridge, UK). This assay is a sensitive and rapid assay to detect G6PDH activity in cells [73]. G6PDH is the rate-limiting step in the pentose phosphate pathway (PPP), catalysing the oxidation of glucose-6-phosphate to 6-phosphoglucono-δ-lactone, leading to the reduction of NADP^+^ to NADPH [74,75]. The PPP is essential for maintaining NADPH and is crucial for redox regulation via the regeneration of GSH. In this assay, G6PDH is oxidised, resulting in the conversion of the colourless probe to a yellow-coloured product, with an absorbance at 450 nm [73].

SH-SY5Y cells were seeded in T-75 flasks and treated with rotenone as previously described. The method was followed according to the manufacturer’s instructions. The absorbance was measured at a wavelength of 450 nm using a Tecan Spark Microplate Reader (Tecan; Zurich, Switzerland).

### 4.13. Quantification of Cellular CoQ_10_ Content

Cellular CoQ_10_ content was assessed in SH-SY5Y human neuroblastoma cells using High-Performance Liquid Chromatography with electrochemical detection (HPLC-ECD). SH-SY5Y cells were seeded in 6-well plates and treated with rotenone as previously described. Samples were rapidly homogenised with 50 µL PBS. Samples were stored at −80 °C and freeze-thawed prior to use. CoQ_10_ was extracted by adding 250 µL of 1-propanol (ratio 1:5) and vortexed. After vortexing, samples were centrifuged at 20,900× *g* for 2 min at 4 °C and 40 µL of supernatant was injected into the HPLC. The chromatographic system used is equipped with a Shiseido Co., Ltd. (Tokyo, Japan) 3005 electrochemical detector (ECD), 2 Shiseido-M 3201 pumps, a Shiseido-M 3023 refrigerated automatic sampler, and a Shiseido-M 3012 switch valve. The total cellular CoQ_10_ content was expressed as µg CoQ_10_/mg of protein [65].

### 4.14. Total Protein Determination

Cellular protein concentration was determined using the method of Lowry et al. [65].

### 4.15. Statistical Analysis

All results are expressed as mean ± standard error of the mean (SEM). Error bars represent SEM. Data were subjected to normality testing using the Shapiro–Wilk normality test to determine the appropriate parametric analysis. A One-Way Analysis of Variance (ANOVA) (groups ≥ 3) or Kruskal–Wallis Test with Tukey’s multiple comparison post hoc test was used to compare the data sets. *p* < 0.05 was considered significant. All experiments were carried out in triplicate and repeated a minimum of 3 times. Statistical analysis was conducted using the GraphPad Prism software (10.2.3) and graphs were created using GraphPad Prism.

## 5. Conclusions

In conclusion, the results from this study are in agreement with previous studies demonstrating the neurotoxic effect of rotenone on cell viability and mitochondrial function in a rotenone-induced neuronal cell model of PD. Rotenone toxicity was shown to induce a loss of cell viability, inhibit the activity of MRC complex I and complex II–III, and inhibit antioxidant defence systems shown by decreased intracellular GSH status and depleted CoQ_10_ levels, in addition to ATP synthesis inhibition. This study further provided evidence demonstrating the effect of rotenone-induced SH-SY5Y cell damage as a result of elevated oxidative stress, indicated by an increase in intracellular and mitochondrial ROS formation. Notably, the association between the effect of G6PDH activity and reduced intracellular GSH synthesis was an interesting finding, which has yet to be shown in an in vitro neuronal cell model of PD. Supplementation with CoQ_10_ was able to prevent increased intracellular and mitochondrial ROS production to control levels in some cases. However, further research is required to fully understand the ability of CoQ_10_ to improve impaired mitochondrial function. Although intracellular and mitochondrial oxidative stress was shown to be attenuated following CoQ_10_ supplementation, treatment may be partially refractory in neurons, as CoQ_10_ supplementation was not effective at reducing ROS levels to that of the control. This may reflect the dosage of CoQ_10_, and higher doses (≥10 µM) may be required to elicit biochemical efficacy. However, these results provide important insights into the potential use of CoQ_10_ as a therapeutic agent in the treatment of PD as a result of its important function in the MRC, its potent antioxidant activity, and its ability to scavenge free radicals. However, this study was carried out using the SH-SY5Y human neuroblastoma cell line; therefore, further research is essential in animal models of PD to establish these mechanisms and to assess the therapeutic efficacy of CoQ_10_ in PD patients in order to validate the findings of our in vitro study.

## Data Availability

The original contributions presented in the study are included in the article, further inquiries can be directed to the corresponding author.
